# Molecular cytogenetic analysis of breast cancer cell lines

**DOI:** 10.1054/bjoc.2000.1458

**Published:** 2000-10-26

**Authors:** J M Davidson, K L Gorringe, S-F Chin, B Orsetti, C Besret, C Courtay-Cahen, I Roberts, C Theillet, C Caldas, P A W Edwards

**Affiliations:** Department of Pathology, University of Cambridge, Tennis Court Road, Cambridge, CB2 1QP; Department of Oncology, University of Cambridge, CIMR, Welcome Trust/MRC Building, Box 139 Addenbrookes Hospital, Hills Road, Cambridge, CB2 2XY, UK; Equipe Genome et Cancer, UMR CNRS 5535, Centre de Recherche en Cancerologie, Val d'Aurelle-Paul Larmarque, Montpellier cedex 5, 34298, France

**Keywords:** chromosome translocations, karyotypes, fluorescence-in situ hybridization, spectral karyotyping, comparative genomicybridization

## Abstract

The extensive chromosome rearrangements of breast carcinomas must contribute to tumour development, but have been largely intractable to classical cytogenetic banding. We report here the analysis by 24-colour karyotyping and comparative genomic hybridization (CGH) of 19 breast carcinoma cell lines and one normal breast epithelial cell line, which provide model examples of karyotype patterns and translocations present in breast carcinomas. The CGH was compared with CGH of 106 primary breast cancers. The lines varied from perfectly diploid to highly aneuploid. Translocations were very varied and over 98% were unbalanced. The most frequent in the carcinomas were 8;11 in five lines; and 8;17, 1;4 and 1;10 in four lines. The most frequently involved chromosome was 8. Several lines showed complex multiply-translocated chromosomes. The very aneuploid karyotypes appeared to fall into two groups that evolved by different routes: one that steadily lost chromosomes and at one point doubled their entire karyotype; and another that steadily gained chromosomes, together with abnormalities. All karyotypes fell within the range seen in fresh material and CGH confirmed that the lines were broadly representative of fresh tumours. The karyotypes provide a resource for the cataloguing and analysis of translocations in these tumours, accessible at http://www.path.cam.ac.uk/~pawefish. © 2000 Cancer Research Campaign


British Journal of Cancer (2000) **83**(10), 1309–1317
© 2000 Cancer Research Campaign
doi: 10.1054/ bjoc.2000.1458, available online at http://www.idealibrary.com on IDEAL®

### Molecular cytogenetic analysis of breast cancer cell lines

**JM Davidson[1], KL Gorringe[2], S-F Chin[2], B Orsetti[3], C Besret[1], C Courtay-Cahen[1], I Roberts[1], C Theillet[3], C Caldas[2] and PAW Edwards[1]**

[1]Department of Pathology, University of Cambridge, Tennis Court Road, Cambridge CB2 1QP; [2]Department of Oncology, University of Cambridge, CIMR, Welcome Trust/MRC Building, Box 139 Addenbrookes Hospital, Hills Road, Cambridge CB2 2XY, UK; [3]Equipe Genome et Cancer, UMR CNRS 5535, Centre de Recherche en Cancerologie, Val d'Aurelle-Paul Larmarque, 34298 Montpellier cedex 5, France

**Summary** The extensive chromosome rearrangements of breast carcinomas must contribute to tumour development, but have been largely intractable to classical cytogenetic banding. We report here the analysis by 24-colour karyotyping and comparative genomic hybridization (CGH) of 19 breast carcinoma cell lines and one normal breast epithelial cell line, which provide model examples of karyotype patterns and translocations present in breast carcinomas. The CGH was compared with CGH of 106 primary breast cancers. The lines varied from perfectly diploid to highly aneuploid. Translocations were very varied and over 98% were unbalanced. The most frequent in the carcinomas were 8;11 in five lines; and 8;17, 1;4 and 1;10 in four lines. The most frequently involved chromosome was 8. Several lines showed complex multiply-translocated chromosomes. The very aneuploid karyotypes appeared to fall into two groups that evolved by different routes: one that steadily lost chromosomes and at one point doubled their entire karyotype; and another that steadily gained chromosomes, together with abnormalities. All karyotypes fell within the range seen in fresh material and CGH confirmed that the lines were broadly representative of fresh tumours. The karyotypes provide a resource for the cataloguing and analysis of translocations in these tumours, accessible at http://www.path.cam.ac.uk/~pawefish. © 2000 Cancer Research Campaign

**Keywords**: chromosome translocations; karyotypes; fluorescence-in situ hybridization; spectral karyotyping; comparative genomic hybridization

The karyotypes of a large proportion of carcinomas are highly abnormal, with many translocated or otherwise rearranged chromosomes and numerical changes (Dutrillaux, 1995; Heim, 1996). This is particularly so for breast carcinomas: in about two-thirds of cases, more than 20% of the chromosomes show structural rearrangements (Dutrillaux et al, 1991). These rearrangements must play some part in the genetic changes that drive carcinoma development, yet we know rather little about them. Classical chromosome banding could not reliably analyse such grossly abnormal karyotypes (Veldman et al, 1997; Macville et al, 1999; Adeyinka et al, 2000). FISH (fluorescence in situ hybridization) techniques, however, now make it possible to identify chromosomes unambiguously and hence obtain accurate karyotypes (Schrock et al, 1996; Speicher et al, 1996; Morris et al, 1997).

We report the application of 24-colour chromosome painting, together with comparative genomic hybridization (CGH), to 20 breast cell lines, 19 from breast cancers and one (HB4a) from SV40-immortalized normal mammary luminal epithelial cells. In 24-colour chromosome painting, each of the 24 human chromosomes is hybridized simultaneously to chromosome paint, but each chromosome is labelled with a different combination of fluorochromes (Schrock et al, 1996; Speicher et al, 1996). CGH complements the karyotyping by showing large-scale gains and losses of chromosomal material, providing an indication of which regions of chromosomes are over- or under-represented in the karyotypes (Macville et al, 1999).

*Received 8 May 2000*
*Revised 5 July 2000*
*Accepted 7 July 2000*

*Correspondence to*: PAW Edwards

### MATERIALS AND METHODS

### Cell culture and metaphase spreads

Cell lines obtained from originators and cultured accordingly were: CAL51 (Gioanni et al, 1990); HB4a (Stamps et al, 1994); MaTu/Ham, MT-1, MT-3 (Hambly et al, 1997) – MaTu/Ham was rederived from a xenograft of the original MaTu of Widmaier et al (1974); SUM-159 (Forozan et al, 1999); VP229 and VP267 (McCallum and Lowther, 1996). Other lines were cultured in 50:50 DMEM/Ham's F12 nutrient mixture, 10% (v/v) FCS, insulin (10 $\mu$g ml$^{-1}$), antibiotics and, for HB4a, hydrocortisone (5 $\mu$g ml$^{-1}$). Lines PMC42 (Whitehead et al, 1983), SK-BR-3 and SK-BR-7 (Fogh and Trempe, 1975; SK-BR-7 was derived by the same authors) were obtained via Dr MJ O'Hare. ATCC lines were obtained via ECACC (European Cell and Animal Culture Collection) (MDA-MB-361, T-47D and ZR-75–1) or Dr O'Hare (MCF-7, MDA-MB-134, MDA-MB-157, MDA-MB-175, MDA-MB-435, ZR-75–30). Second samples of MaTu and MT-1 were obtained for authentication from Dr I Fichtner, MDC, Berlin, the laboratory where xenografts that gave rise to MaTu/Ham and MT-1 were derived (Naundorf et al, 1993).

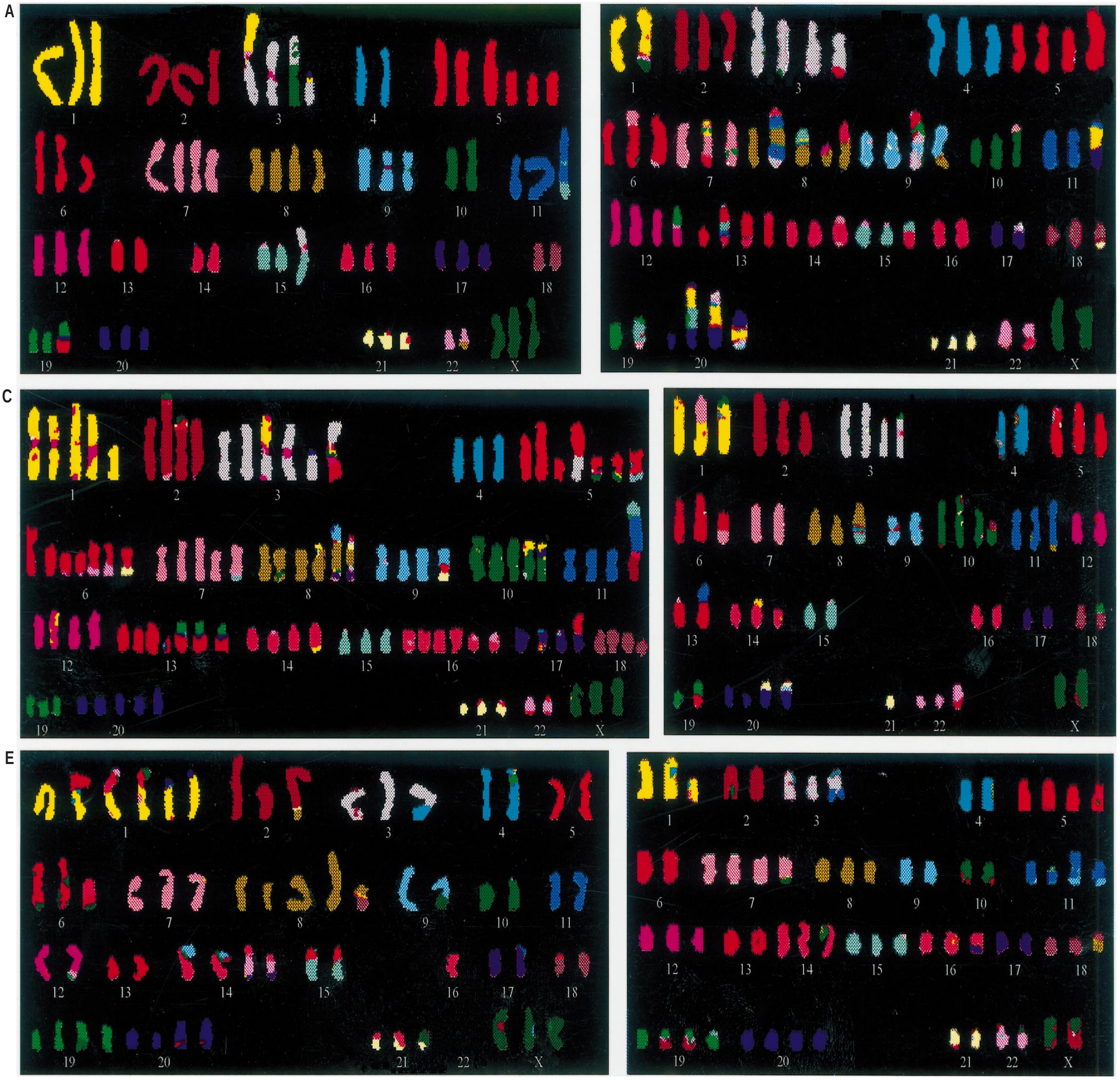

### Fluorescence in situ hybridization1

24-colour chromosome painting was performed by the 'spectral karyotyping' (SKY) technique, essentially as described (Schrock et al, 1996; Roberts et al, 1999). Briefly, metaphases were hybridized with chromosome paints for all the chromosomes simultaneously, each chromosome being labelled directly or indirectly with a distinct combination of up to five fluorochromes: fluorescein, Spectrum Orange, Texas Red, Cy5 and Cy5.5. These chromosome paints were either from Applied Spectral Imaging (Migdal HaEmek, Israel) or as described (Roberts et al, 1999). Metaphases were imaged on a fluorescence microscope to show all five fluorochromes simultaneously, and the fluorescence analysed using a spectrometer and CCD camera (Spectracube, Applied Spectral Imaging). DNA was counterstained and imaged separately with DAPI (4,6-diamino-2-phenylindole). The supplied software analyses the spectrum of each pixel of the image, determines which dyes are present and hence which chromosome. Each pixel of the image is then presented false-coloured in a 'classification colour' to show which chromosome spectrum best matches the spectrum recorded, as in Figure 1.

Ten metaphases were analysed by 24-colour fluorescence, except for MCF-7, and in addition chromosome copy numbers and translocated fragments were verified in a number of additional metaphases analysed by conventional 2- or 3-colour chromosome painting using separate fluorescent dyes for each chromosome paint. Representative karyotypes (Table 1) were assembled as follows: all rearranged chromosomes present in at least two metaphases were listed, and the number of copies of each chromosome given is the mode, except where noted. The metaphases illustrated in Figure 1 show all the abnormal chromosomes present in two or more metaphases. They comprised one typical metaphase, with additional chromosomes from other metaphases where that chromosome was absent from the chosen metaphase,

 © 2000 Cancer Research Campaign

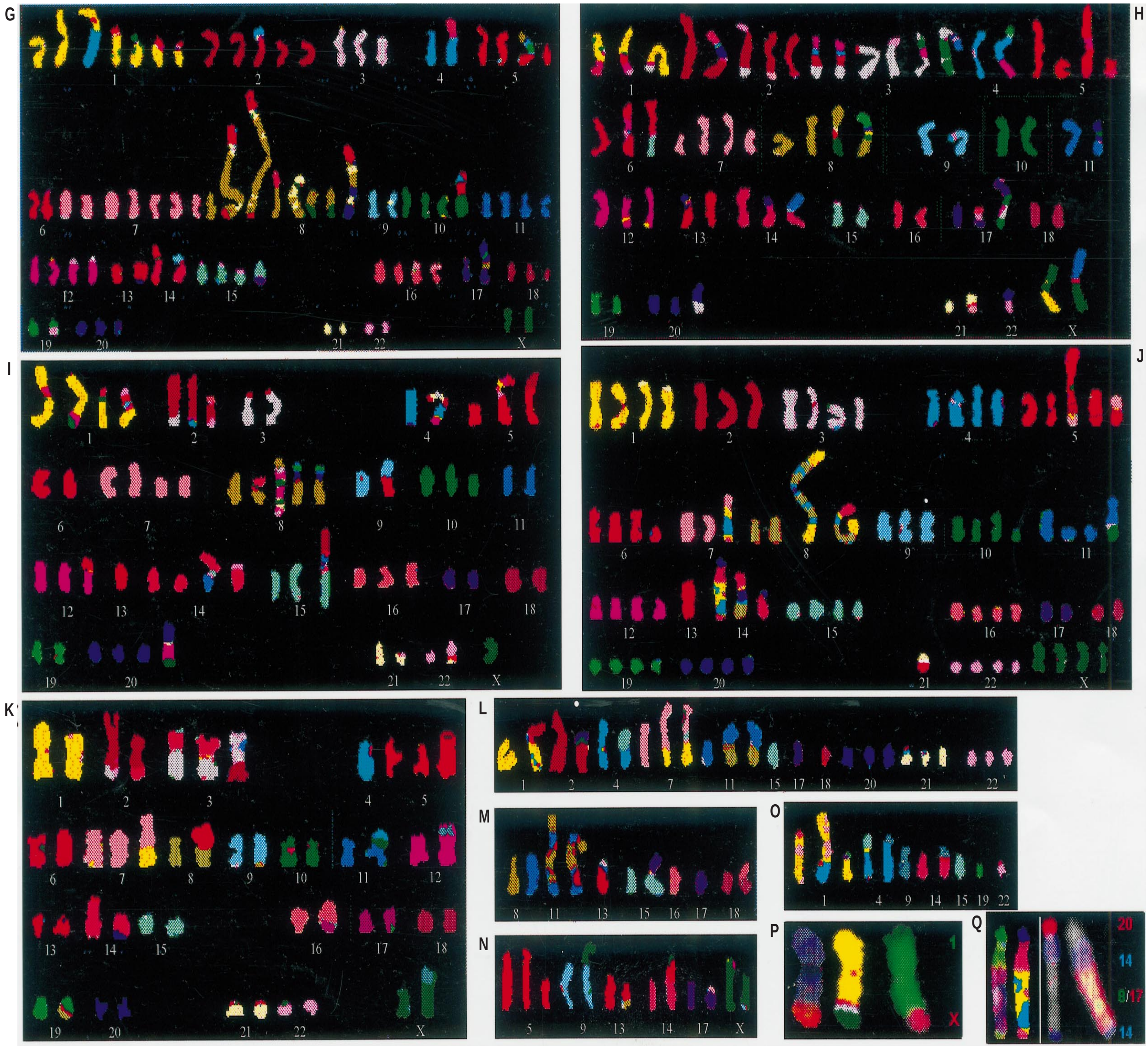

**Figure 1** 24-colour karyotypes. These two plates show the full representative karyotypes of the more complex lines (except the three lines analysed previously by reverse painting, MDA-MB-361, T-47D and ZR-75-1, which are illustrated in more detail in Figures 6–8 of Morris et al (1997)); the deviations from diploidy for the lines with near-diploid karyotypes (except CAL51 and MT-3); and examples of analysis of individual chromosomes showing how the 24-colour analysis is completed by conventional chromosome painting. In the karyotype images, the chromosomes are shown in classification colours (see text). Some of these classifications are incorrect in detail, as illustrated in P and Q, and the correct identifications are listed in Table 1. **A** MaTu/Ham; **B** MCF-7; **C** MT-1; **D** MDA-MB-435; **E** MDA-MB-157; **F** PMC42; **G** SK-BR-3; **H** VP229; **I** VP267; **J** ZR-75-30; **K** HB4a. **L** to **O** deviations from diploid for near-diploid lines – all copies, both translocated and apparently normal, of the chromosomes affected by rearrangement are shown: **L** MDA-MB-175; **M** MDA-MB-134; **N** SUM-159; **O** SK-BR-7. In **M** the 8;11 translocation has an hsr that is a coamplification of material from chromosomes 8 and 11 (Bautista & Theillet, 1998) much of which is misclassified as chromosome 13. **P, Q** examples of the completion of analysis of aberrant chromosomes by conventional chromosome painting. In both, the first image is an RGB representation of the fluorescence of the chromosome; the second image is the classification of this fluorescence by the software, and the subsequent images are copies of this chromosome from a conventional chromosome painting experiment. **P** 1;X translocation on MCF-7, showing artefactual identification of strips of chromosomes 3 and 6 in the region where the fluorescence of the 1 (stained with dyes BCD) and X (dyes AE) fragments overlap to create spectra expected for 3 (dyes ACDE) and 6 (dyes BCDE); and two-colour hybridization with 1 (green) and X (red) paints. Chromosome 3 and 6 paints did not hybridize (not shown). **Q** der (?)t(20;14;(8;17)$_n$;14) of ZR-75-30. A region of this chromosome is made up of interleaved small pieces of chromosomes 8 (dye D) and 17 (dye C), whose blended fluorescence was classified as chromosome 4 (dyes CD) and, where close to chromosome 14 material (dye B), as chromosome 1 (dyes BCD).

scaled where necessary. As a result the number of copies of chromosomes shown in this metaphase is not necessarily the same as the (modal) number given in Table 1.

Individual chromosome paints for conventional chromosome painting, and centromeric repeat probes (prelabelled, from Oncor), were labelled and hybridized as described (Courtay-Cahen et al, 2000). For 3-colour analysis FITC, Spectrum Orange (or Cy3) and Cy5 were used.

Comparative genomic hybridization (CGH) was done essentially as described (Kallioniemi et al, 1994; Courjal and Theillet,

**Table 1** Karyotypes of the 20 breast cell lines

**CAL51** (ISCN notation)[a] 46(45–46), XX

**MaTu** 69(62–70). 1×3, 2×2, del(2)(q?), der(3)t(1;3)(q22;?), der(12?)ins(3;12), der(3)t(3;10), der(3)t(3;20), 4×2, 5×3, i(5)(p)×3, 6×2, i(6)(p), 7×4, 8×4, 9×3, 10×2, 11×1, der(11)dup(11)(?), der(11)t(11;15)(q21;?), 12×3, 13×2, 14×2, 15×2, der(15)t(3;15), 16×3, 17×3, 18×2, 19×1, dup(19), der(19)t(13;19)(q12;?), 20×3, 21×3, 22×1, der(22)t(8;22)(q23–24.2;?), X×3 cp[10]

**MCF-7** 65 (61–72).1×1, der(1)t(X;1), 2×2, der(2)t(2;3)(q36;?), 3×2, del(3)(p?), der(3)t(3;6), 4×3, 5×3, der(5)t(5;13)(p12?;q22), 6×1, der(6)t(6;7), der(6)t(3;6), 7×1, der(7)t(1;19;7;6), der(7)t(7;19;7), 8×1, der(?)t(16;11;8;11;3), der(8)t(8;15), der(8?)t(8;12), der(8)t(8;16), 9×2, der(?)t(6;20;9;3), der(9)t(8;9), 10×2, der(10)t(7;10)(?;q22), 11×2, der(?)t(11;1;17;19;17), 12×3, der(19)t(12;19), del(13)(q?), der(13)t(13;15;11;16), der(13)t(13;14), der(13)t(13;16), 14×2, der(?)t(7;14), 15×2, der(16)t(15;16), 16×2, 17×1, der(17)t(3;17), 18×2, der(18)t(18;21), 19×1, der(19)t(7;19), 20×1, der(?)t(17;1;19;17;20), der(20)t(3;20;1;20;1;20), der(20)t(20;1;21), 21×3, der(22)t(7;22), der(22)t(16;22), X×2 cp[6]

**MDA-MB-134** 44 (37–47) and 66(60–77).1×2, 2×2, 3×2, 4×2, 5×2, 6×2, 7×2, 8×1, der(?)t(8;11)ins(11;8)$_n$×2, 9×2, 10×2, 11×1, 12×2, 13×1, 14×2, 15×1, der(?)t(15;17), 16×1, 17×1, 18×1, der(18)t(16;18), 19×2, 20×2, 21×2, 22×2, X×2 cp[8]/idem×2, –1, –5, +16×2, –19, –X, –der(?)t(8;11)ins(8;11)$_n$, –der(18)t(16;18), –der(?)t(15;17) [7]

**MDA-MB-157** 62 (41–68) and 116(101–120).1×1, der(1)t(1;13)(?;q21), der(1)t(5;16;1)×2, der(1)t(1;20)×2, 2×1, del(2)(p?), der(2)t(2;8), 3×2, der(3)t(3;4), 4×2, 5×2, 6×2, der(16)t(6;16), 7×3, 8×1, del(8)(p?), i(8)(q?)×2, der(?)t(8;18), 9×1, der(9)t(X;9), 10×2, 11×2, 12×1, der(12)t(12;19), 13×1, i(13)(q), der(14)t(9;14)×2, der(?)t(14;22;17)×2, der(5)t(5;15)×2, 16×1, 17×2, 18×2, 19×2, der(19)t(10;19)×2, 20×2, der(20)ins(20;13)×2, der(21)t(16;21)×2, der(21)t(X;21), X×3 cp[5]/idem, – der(1)t(1;13), + del(1)(q31)×2 [5]/idem×2, +del(8)(p?), –i(13)(q)×2, –19, +der(19)t(10;19), –der(20)ins(20;13)×2, +21×2, –der(21)t(16;21), –X [6].

**MDA-MB-175** 48 (43–48).1×1, der(1?)t(1;2), 2×1, der(2)t(2;17), 3×2, 4×1, der(4?)t(4;15), 5×2, 6×2, 7×1, dic(1;7)t(1;7)×2, 8×2, 9×2, 10×2, 11×1, der(8?)t(8;11)×2, 12×2, 13×2, 14×2, 15×1, 16×2, 17×1, 18×1, 19×2, 20×3, 21×3, 22×3, X×2 cp[10]

**MDA-MB-361** 51(43–57).1×1, del(1)(p31.1), 2×2, del(2)(p?), 3×2, der(3)t(3;7;13), 4×2, der(4)t(1;4), 5×1, i(5)(p)?, der(?)t(5;20), 6×2, der(6?)t(6;12;15;22), 7×2, 8×1, der(8)t(8;17;8), der(8)t(8;17;15), 9×1, der(?)t(9;11), 10×2, der(10)t(1;10), der(11)t(2;11), der(?)t(11;16), 12×3, 13×1, der(?)t(13;21), 14×1, der(X)t(14;18;X), der(14)t(14;16), der(?)t(X;15), 16×1, der(16)t(X;16), der(16)t(12;16), der(?)t(16;20), der(16)t(16;17), der(?)t(7;17), der(17)t(16;17), der(17)t(9;17), 18×2, der(?)t(18;20), 19×2, del(20)(p?), 21×1, 22×2, X×1 cp[10]

**MDA-MB-435** 57(44–62).1×1, der(1)t(1;7), der(1)t(1;10), 2×2, del(2)(p?), 3×2, del(3)(p?), der(3)t(3;19), 4×2, 5×2, del(5)(q?), 6×2, der(6)t(6;7), 7×2, 8×1, del(8)(p), der(8)t(8;15), 9×2, 10×2, del(10), der(10)t(10;18), 11×2, der(11)t(8;11), 12×2, 13×1, der(13)t(11;13), 14×1, der(14)t(1;14), der(?)t(14;16), 15×2, 16×2, 17×2, 18×1, der(?)t(18;19), 19×1, der(?)t(6;19), 20×1, del(20), der(?)t(20;21)×2, 21×1, 22×2, der(22?)t(6;22), X×2 cp[10]

**MT-1** 103(90–103).1×4, del(1), 2×1, der(2)t(X;2;7), der(2)t(X;2), der(2)t(2;18), 3×2, der(3)t(1;3), der(12)ins(3;12), der(3)t(3;20), der(?)t(3;1;6), 4×3, 5×1, dup(5)(p?), i(5)(p), der(5)t(3;5), der(5)t(5;10)×2, der(5)t(5;15), 6×1, del(6)×2, der(6)t(6;22)×2, **der(6)t(6,21)**[b], 7×2, i(7)(q), del(7)(q?), der(7)t(7;15), 8×4, der(8)t(9;8;17), der(8)t(6;8;17), 9×3, der(9)t(9;21), 10×3, der(10)t(1;10)×2, 11×3, der(2)t(15;11;2), 12×3, 13×3, del(13), der(19)t(13;19)×2, der(19)t(13;19), 14×3, der(14)t(1;14), 15×3, 16×4, del(16)×2, 17×3, der(17)t(5;17), 18×3, del(18), 19×3, 20×5, 21×1, **der(21)t(6;21)×2**, 22×1, der(22)t(8;22), X×3 cp[10]

**MT-3** (ISCN notation)[a] 46(36–47), X, +7, i(13)(q), –X [10]

**PMC42** 57(52–67).1×2, del(1)(q?), 2×1, del(2)(p?), 3×1, del(3)(p?), der(3)t(3;11), 4×2, 5×4, 6×2, 7×3, der(7)t(7;10), 8×2, del(8)(q?), 9×2, 10×2, 11×1, del(11)(p?), der(11)t(9;11)×2, 12×3, 13×2, 14×2, der(14)t(10;14), 15×3, 16×2, der(16)t(16;17), 17×2, 18×2, der(?)t(8;18), 19×1, der(19)t(2;19)×2, der(19)t(15;19), 20×4, 21×2, 22×2, X×1, der(X)t(X;2) cp[10]

**SK-BR-3** 79(68–79). 1×1, i(1)(q), der(1?)t(1;4), der(1)t(1;5), der(1)t(1;10), der(1)t(1;5), del(1)?, 2×2, der(?)t(2;6;4), der(2)t(2;6)×2, 3×2, del(3), 4×1, der(?)t(4;14), 5×2, der(?)t(5;19;4;8), del(5), 6×2, 7×7, 8×1, der(8?)t(13;3;8;3;8;13)×2, der(?)t(8;14), der(8)t(8;21), der(?)t(X;8)×2, der(?)t(20;3;8;17;19;8;3;13), 9×2, 10×1, del(10)×2, der(10?)t(6;4;6;10), 11×4, 12×2, der(12)t(3;12)(?;q23)×2, 13×1, der(13)t(13;20), der(14)t(5;14), der(?)t(9;14), 15×2, del(15), der(15)t(15;17), 16×2, der(16)t(7;16)×2, 17×1, der(?)t(20;19;8;17), 18×2, del(18), 19×1, der(?)t(19;22), 20×3, 21×3, 22×2, X×2 cp[10]

**SK-BR-7** 43(38–44).1×1, der(1)t(1;4), der(?)t(1;19), 2×2, 3×2, der(15?)t(4;15), der(4)t(4;9), 5×2, 6×2, 7×2, 8×2, 9×1, 10×2, 11×2, 12×2, 13×2, 14×1, der(9)t(9;14), 15×1, 16×2, 17×2, 18×2, 19×1, 20×2, 21×2, 22×1, X×2 cp[10]

**SUM-159** 47(46–59). 1×2, 2×2, 3×2, 4×2, 5×2, del(5)(q11.2), 6×2, 7×2, 8×2, 9×1, **der(9)t(X;9)**, 10×2, 11×2, 12×2, 13×1, der(13)t(8;13), der(?)t(14;13;8), 14×1, 15×2, 16×2, 17×1, der(17)t(7;17)(p22;?), 18×2, 19×2, 20×2, 21×2, 22×2, X×1, **der(X)t(X;9)** cp[10]

**T-47D** 62(44–66).1×2, der(16)t(1;16), 2×2, 3×2, del(3)(p?), der(?)ins(3;5), 4×2, der(?)t(4;5), 5×2, 6×2, 7×2, der(7)t(7;15)×2, 8×2, der(8)t(8;14)×2, der(9)t(9;17)×2, der(10)t(10;20), der(10)t(10;20), der(10)t(3;10)×2, del(10)×2, 11×2, del(11)(p?)×2, 12×1, der(12)t(12;16), der(12)t(12;13), del(12)(p?), 13×2, 14×2, 15×1, der(?)ins(9;15)×2, 16×2, 17×2, 18×2, del(18), 19×2, 20×2, 21×2, 22×2, X×1, der(X)t(X;6) cp[10]

**VP229** 62(56–66). der(1)t(1;14), der(X)t(X;1), der(?)t(1;9;10?;22), der(1)t(1;3), 2×1, der(?)t(14;20;2)*, **der(2)t**(22;**2;3)**, der(2)t(2;7), der(2)t(2;14)*, der(2)t(12;4;2;3;2)×2, **der(3)t(2;3)**, der(3)t(3;22), der(3)t(10;12;10;12;3), der(3)t(11;3;10;1?)*, 4×1, der(4)t(19;4;X;12), 5×1, del(5)(q?), der(5)t(5;6), der(5)t(5;14), 6×2, der(6)t(6;15), 7×2, del(7)(q?)×2, 8×2, der(8)t(8;X), der(8)t(19;1;8), 9×1, der(9)t(4;9), 10×2, 11×1, der(?)t(20;3;11)*, der(X)t(X;11), 12×1, der(?)t(12;?)×2, 13×1, der(13)t(20;13), i(14)(q?), der(?)t(14;17?), der(14)t(11;14), 15×2, 16×2, 17×1, der(?)t(16;17), der(17)t(17;10;3;10;3;10), 18×2, 19×2, 20×2, der(20)t(20;3), 21×1, der(21?)t(21;7), der(?)t(20;22) cp[10]

**VP267** 59(50–59) der(1)t(1;9?;10;1), der(1)t(1;12;10;12), der(1)t(1;14), der(?)t(1;9;10?;22), **der(2)t(2;3)**, der(2)t(2;4), der(2)t(16;2;22), **der(3)t(2;3)**, der(3)t(2;3), 4×1, der(4)t(2;3;2;4;12), del(5)(q?), der(5)t(5;1;4;3;6), der(5)t(2;5), 6×1, der(6), 7×2, del(7)×2, 8×1, der(8)t(8;14)*, der(8?)t(8;3;4;10;3;4?;10;4?;10?)?, der(8)t(8;19)×2, 9×1, der(9)t(9;13), 10×2, del(10), 11×2, 12×2, der(12)t(6;12), 13×1, 14×1, del(14), der(14)t(11;14), der(14)t(14;22), 15×1, i(15), der(15)t(15;4;11;2), 16×2, der(16)dup(16)?, 17×2, 18×2, 19×2, 20×3, der(20)t(20;?;12;X), 21×1, der(21)t(21;22), 22×1, der(22)t(21;22), X×1 cp[10]

**ZR-75–1** 72(51–75). 1×2, der(1)t(1;12)(?;q14), del(1)(p?)hsr(1)?, 2×3, 3×3, 4×3, 5×3, 6×1, del(6)(q?)×2, 7×3, der(22)t(7;22)×3, 8×3, der(8)t(8;11)(p12;?), 9×3, 10×3, der(11)t(11;17)×2, 12×3, der(?)t(12;16), der(?)t(1;12), 13×2, 14×2, der(14)t(13;14), 15×2, der(15)t(10;15), der(15)t(1;15), 16×1, i(16)(q), i(16)(p), 17×2, der(?)t(6;17), 18×4, 19×2, del(19)(p?), 20×2, i(20)(q), 21×2, 22×2, der(22)t(15;22)*, X×3 cp[10]

**ZR-75–30** 79(76–80).1×2, der(1)t(1;21)(p34.1;?)×2, 2×3, 3×4, 4×4, 5×2, der(5)t(5;13;7), der(5)t(5;13), der(5)t(7;5), 6×3, del(6)(q25), 7×2, der(7)t(7;(8;17)$_n$;14), 8×2, der(?)t(1;17;8;17;8;17;8;1), der(?)t(14;(8;17)$_n$;1), 9×3, 10×3, **der(10)t(11;10)**, 11×1, del(11)(q?)×2, **der(11)t(11;10)**, 12×4, i(13)(q)*, der(?)t(20;14;(8;17)$_n$;14), der(?)t(14;17;8), der(14)t(20;14), 15×4, 16×4, 17×2, 18×2, 19×4, 20×4, der(21)t(21;13), 22×4, X×4, cp[10]

**HB4a** 42(35–56) and 69(63–87). 1×1, del(1)(p33), **der(2)t(2;3)**, der(2)t(2;6), der(3)t(3;14)(?;q24.1)×2, **der(3)t(2;3)**, 4×1, 5×2, der(5)t(2;5), 6×2, 7×2, der(7)t(1;7), 8×1, der(8)t(8;13), 9×1, der(9)t(8;9), 10×2, 11×1, der(11)t(4;X?;11), 12×1, der(12)t(9;12)(?;p12.1), 13×2, i(14)(q), der(14)t(14;20), 15×2, 16×1, der(16?)t(16;17), 17×1, der(17)t(17;19), 18×2, 19×1, der(19)t(8;19), 20×2, 21×2, 22×2, X×1, der(?)t(X;9) cp[6]/idem, –i(14), +14, –der(X?)t(X;9), +der(X?)t(5;X;9) [4]/1×2, del(1)×2, der(2)t(2;3)×2, der(2)t(2;6)×2, der(3)t(3;14)×2, der(3)t(2;3)×2, 4×2, 5×3, der(5)t(2;5), 6×4, 7×4, der(7)t(1;7)×2, 8×3, der(8)t(8;13)×2, 9×1, der(9)t(8;9), 10×2, 11×2, der(11)t(4;X?;11)×2, 12×1, der(12)t(9;12)×2, 13×4, i(14)×2, der(14)t(14;20)×2, 15×4, 16×2, der(16?)t(16;17)×2, 17×3, der(17)t(17;19), 18×4, 19×2, der(19)t(8;19)×2, 20×4, 21×4, 22×4, X×2, der(?)t(X;9)×2 [4]

With the exception of CAL51 and MT-3, karyotypes are given as mode (range) then a complete list of chromosomes present. (This is a departure from cytogenetic convention, ISCN 1995, where chromosome numbers are given as changes from the nearest ploidy, since this can be difficult to interpret with these highly rearranged karyotypes). Chromosomes are listed in the order that they appear in Figure 1. The copy numbers of each chromosome are the mode, except that all chromosomes present in more than 2/10 metaphases are included and marked with an asterisk if present in less than half the metaphases. Structural changes are described according to standard (ISCN 1995) nomenclature. Co-amplifications of chromosome fragments with an unknown number of copies of the co-amplification are indicated as (b;c)$_n$. Translocations were verified by single-dye conventional chromosome painting, except for some in cell lines VP229 and VP267. Obvious deletions and isochromosomes are noted together with a few breakpoints that were identified as boundaries of CGH changes and independently by DAPI banding. Clearly distinct clones are indicated in standard nomenclature: they are separated by /, with the number of metaphases examined in square brackets. ‘cp’ indicates that the karyotype given is a composite from a number of slightly differing metaphases. [a]The karyotypes of CAL51 and MT-3 were respectively normal diploid and near-diploid, so are given as deviations from diploid according to ISCN convention, i.e. mode (range), sex chromosome content, numerical and structural changes from diploid. [b]Translocations that may be reciprocal are marked in bold.

*© 2000 Cancer Research Campaign*

1997). Briefly, normal genomic DNA and cell line DNA were labelled with biotin and digoxigenin respectively by DOP-PCR (Telenius et al, 1992). Cot-1 DNA (GibcoBRL) was added and the paints hybridized to normal male metaphases (Vysis). The signals were detected with avidin-TexasRed and FITC-conjugated anti-digoxigenin antibody. The slides were mounted with Vectashield (Vector Labs) containing DAPI. At least 15 metaphases were captured for each cell line on a Zeiss Axioplan II fluorescent microscope using SmartCapture VP software and analysed by Quips CGH Karyotyper and Interpreter software (Vysis); or on a Leica DMRB Microscope, captured using a Kappa CF 8/1 DXC CCD camera integrating in 256 grey levels, using Starwise software from IMSTAR (Paris, France). The threshold set for gains corresponded to a mean hybridization ratio between tumour and normal of >1.2:1, and for losses of <0.8:1.

### RESULTS AND DISCUSSION

We identified the translocations and numerical changes of the chromosomes of the 20 cell lines by 24-colour chromosome painting. As a precaution we confirmed the identity and number of virtually all the translocations by conventional chromosome painting, since in certain circumstances the identification provided by the 24-colour software can be unreliable, as discussed below.

The resulting karyotypes are listed in Table 1. Most are illustrated in Figure 1, which shows complete or partial representative metaphases, false-coloured to show the pixel-by-pixel identification of chromosome material.

### Validation of the 24-colour karyotypes

Identifying chromosomes by marking them with combinations of fluorescence colours works well except where fluorescence from adjacent chromosome fragments blends to create the colour mixture of an irrelevant chromosome. This can lead to misidentification of complex and small rearrangements, as illustrated in Figure 1 P, Q. The simplest case is at the breakpoint of a translocation, where blending of the neighbouring colours suggests the presence of a thin band of a third chromosome, or even of more than one other chromosome. Figure 1 P shows a 1;X translocation in MCF-7. At the junction between the 1 and the X the spectrum blends to match the spectra of chromosomes 3 and 6. A series of conventional chromosome painting hybridizations confirmed that 1 and X were indeed present, while 3 and 6 were absent. Most difficult to identify is where multiple fragments of two or more chromosomes are interleaved, as commonly occurs in hsrs (cytogenetic homogeneously staining regions) in breast carcinomas. Figure 1 Q shows an example from ZR-75-30 where an hsr composed of interleaved fragments of chromosome 8 and 17 is classified as chromosome 4 and, where there is also overlap from the chromosome 14 fragment, as chromosome 1.

We were able to test whether the final karyotypes were reliable because we had previously karyotyped completely three of the lines, MDA-MB-361, T-47D and ZR-75-1, by reverse chromosome painting (Morris et al, 1997): the present karyotypes, obtained without reference to the earlier work, were completely in agreement, except for two trivial discrepancies: we did not identify a der(22) reported in ZR-75-1 by Morris et al (1997) nor the very small marker M20 in T-47D.

### Nature of the karyotypes and comparison with primary material

The carcinoma karyotypes fell into different groups. CAL51 was perfectly diploid, as previously reported by classical banding (Gioanni et al, 1990). MT-3 showed minimal deviation from diploidy – iso13 q, trisomy 7 and loss of X. The iso13q and trisomy 7 – both readily detected by CGH as the sole abnormalities – are typical of near-diploid carcinoma karyotypes and CGH pattern (Dutrillaux, 1995). Two of the lines, MDA-MB-134 and SK-BR-7, were hypodiploid, showing the net loss of chromosomes discussed below. Most of the remainder fell in the highly rearranged, near-triploid range that is typical of a high proportion of breast cancers. Finally, there were three lines that were somewhat different. SUM-159 and MDA-MB-175 had a relatively simple karyotype, near diploid but with a slight net gain of chromosomes compared to diploid. MT-1 was unique in being hypertetraploid and the presence of duplicated abnormal chromosomes strongly suggests that it has endoreduplicated its chromosomes. This suggests that it may represent the result of endoreduplication of a slightly hyperdiploid line like SUM-159 or MDA-MB-175.

The range of karyotypes shown by these lines – the number of rearranged chromosomes as a function of the number of chromosomes – was within that of a large series (113) of freshly-isolated carcinomas surveyed by Dutrillaux et al (1991). However, there were no lines with highly rearranged karyotypes but fewer than 46 chromosomes, a subset that formed almost 40% of the samples of Dutrillaux et al. Since such isolates usually also contain endoreduplicated (i.e. hypotetraploid) variants, one possible explanation is that there is more selective pressure in culture than in vivo for tetraploidization, and such cases yield subtetraploid lines.

### CGH

CGH (Figure 2) provides different information from 24-colour karyotyping, and also can be used to compare the cell lines with uncultured tumours, since it is equally applicable to cultured cells and surgical material (e.g. Persson et al, 1999).

CGH complements 24-colour karyotyping in two ways. It shows deletions and amplification of relatively small regions of chromosomes, which karyotyping generally misses. But also, it shows large scale net chromosome copy number changes, which correlate well with the numerical changes seen by 24-colour karyotyping, and may be used in favourable cases to identify which chromosome fragments are present in the unbalanced translocations (Macville et al, 1999). For example, MaTu has a mode of 69, so a CGH red:green hybridization ratio of 1.0 corresponds to three copies of a chromosome segment. MaTu has three apparently normal chromosome 1s and a 3;1 translocation: CGH gives a ratio of 1.0 for 1p, but around 1.2 for most of 1q, suggesting that the translocation consists of distal 1q. Chromosome 4 is present in 2 copies and has a ratio of around 0.8 for all of its length. There are three apparently normal 5s and three small 5 fragments: the ratio for 5q is 1.0 while 5p is present in considerable excess, showing that the short 5s are 5p, the size suggesting iso5p. Thus where the two techniques can be compared, there is very good agreement, and where CGH shows differences within a chromosome, this can be used to identify the components of a translocation, provided the rearrangements are not too complex, and provided the cell line has one predominant clone.

© 2000 Cancer Research Campaign

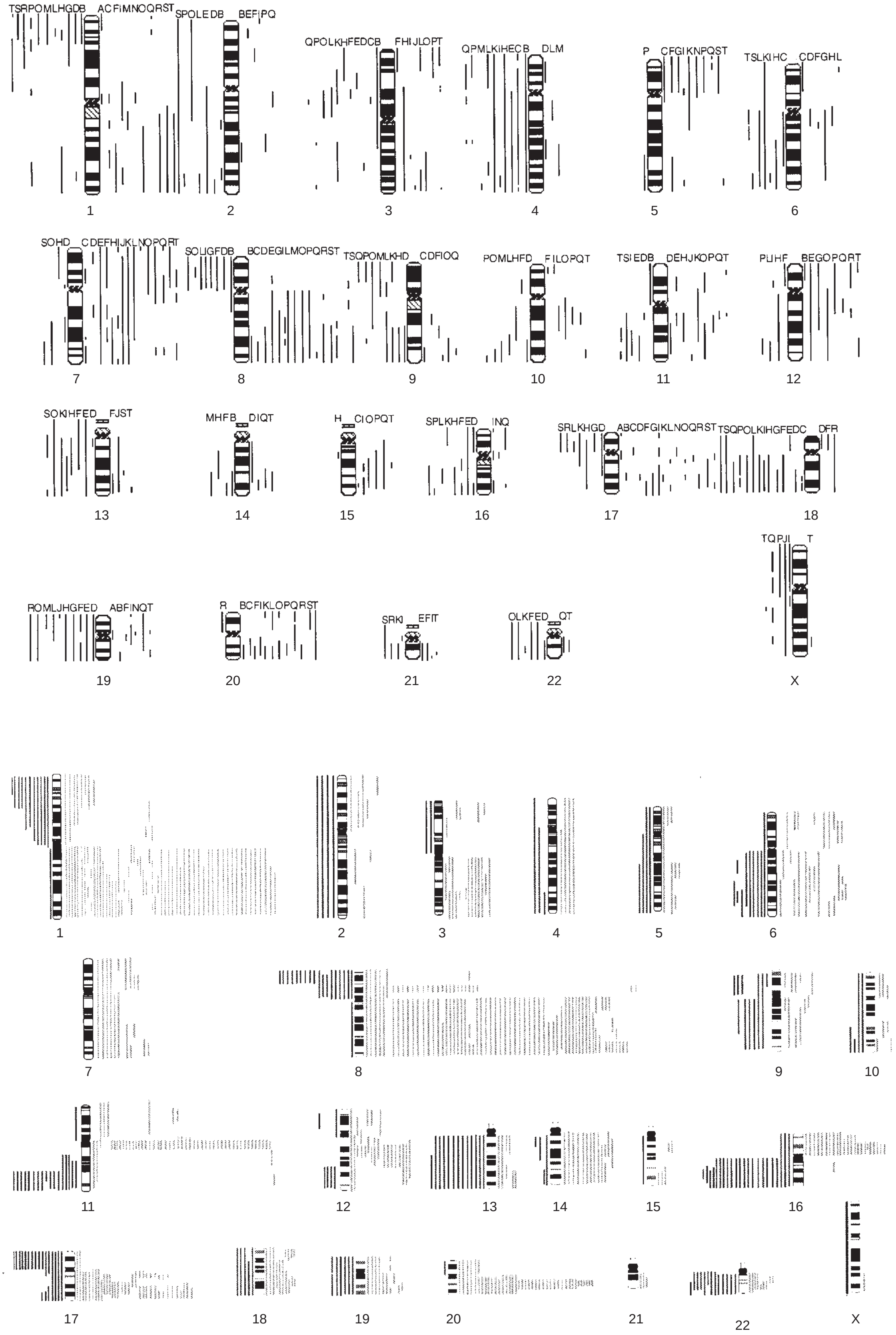

**Figure 2** CGH profiles of breast cell lines and breast cancers. Gains are to the right of each ideogram and losses to the left. Above: Cell lines, (**A**) CAL51, (**B**) HB4a, (**C**) MaTu, (**D**) MCF-7, (**E**) MDA-MB-134, (**F**) MDA-MB-157, (**G**) MDA-MB-361, (**H**) MDA-MB-435, (**I**) MT-1, (**J**) MT-3, (**K**) PMC42, (**L**) SK-BR-3, (**M**) SK-BR-7, (**N**) SUM-159, (**O**) T-47D, (**P**) VP229, (**Q**) VP267, (**R**) ZR-75-1, (**S**) ZR-75-30, (**T**) MDA-MB-175. Below: 106 fresh cases, described in (Courjal and Theillet, 1997; Bautista and Theillet, 1998).

*© 2000 Cancer Research Campaign*

Figure 2 shows that overall the genetic anomalies in breast cancer cell lines are good representations of aberrations arising in uncultured tumours. It compares the CGH profiles for the cell lines with the gains and losses found in 106 breast tumours analysed by us (Courjal and Theillet, 1997; Bautista and Theillet, 1998), which are in general agreement with the survey of Tirkkonen et al (1998). For example, the most frequent gains and losses seen in the tumours are well represented in the cell lines: gains of 1q 8q, proximal 11q and 20q, and losses of 8p, distal 11q, and16q.

### Translocations

There was an enormous range of chromosome abnormalities in these carcinoma lines. All chromosomes were translocated at least occasionally, and they were translocated to a wide range of partners. No translocation appears to be present in a substantial proportion of the lines. The most frequent in the carcinoma lines were 8;11 in five lines; and 8;17, 1;4 and 1;10 in four lines.

Chromosome 8 was overall the chromosome that was involved in the most translocations; it was present in a total of 39 distinct translocated chromosomes in 19 of the cancer cell lines, and was translocated to all other chromosomes except 5, 10 and 20. Many of the breakpoints may of course be completely unrelated, but we have previously shown that some are clustered. The lines MDA-MB-361, T-47D and ZR-75-1 all have a translocation breaking within at most 1.5 Mb on 8p12, with loss of distal 8p, as often seen in carcinomas (Morris et al, 1997; Courtay-Cahen et al, 2000), and the 8;11 of MDA-MB-175 has a breakpoint within about two to four megabases of these, within the *HGL* gene (Wang et al, 1999). Chromosome 13 also has clustered breaks at 13q12, proximal to *BRCA2*, and at 13q14, near to *RB-1* (S-F. C. and C.C., unpublished).

Translocations were almost all unbalanced, as expected for carcinomas (Dutrillaux, 1995). Possible exceptions which might be reciprocal, merely on the basis of appropriate size pieces and centromere position where it is known, were the 6;21 in MT-1, the X;9 of SUM-159, the 2;3 of VP229 and VP267, the 10;11 of ZR-75–30, and the 2;3 in the normal breast line HB4a. This is not more than 6 examples out of a total of 341 translocated chromosomes, or less than 2%.

Several of the cell lines showed complex multiply-translocated chromosomes, for example the 6;12;22;15 in MDA-MB-361 and several in SK-BR-3, MCF-7, VP229, VP267 and ZR-75–30. Many of these complex rearrangements included a coamplification of segments of two chromosomes, as commonly occurs in breast cancers and often gives rise to a cytogenetic hsr (Bautista and Theillet, 1998; Bernardino et al, 1998). MDA-MB-134 had a coamplification of 8 and 11 previously described (Bautista and Theillet, 1998); SK-BR-3 had complex coamplifications of (principally) 8, 3 and 13; VP229 and VP267 had several involving (principally) 3,4,8,10 and 12; ZR-75–30 had alternating 8 and 17. Not all such coamplifications are likely to be resolved by the 24-colour chromosome painting – for example we have shown elsewhere that the 8;17 translocations of MDA-MB-361 have alternating fragments of 8 inserted in the chromosome 17 fragment (Courtay-Cahen et al, 2000). The frequent presence of 8 in these structures has been noted before (Bernardino et al, 1998; Courtay-Cahen et al, 2000).

### Karyotype patterns and their development

The question of how a karyotype has evolved could be important because it may reveal the events that lead to translocation or numerical changes, and the nature of the selective pressures operating on the resulting gene changes. Dutrillaux and colleagues (Muleris et al, 1988; Dutrillaux, 1995) described two main modes of karyotype evolution, designated ‘trisomic’ and ‘monosomic’. The trisomic type seemed to gain extra chromosomes, while the monosomic type tended to lose a chromosome when a translocation occurred. MT-3, with trisomy 7 and iso 13 q is a very simple trisomic case. MDA-MB-134 is an example of monosomic pattern: an 8 and an 11 have been replaced by an unbalanced translocation der(?)t(8;11); a 15 and a 17 have been replaced by a der(?)t(15;17); and a 16 and an 18 by a der(18)t(16;18). These have presumably resulted in net losses of one copy (allele) of part of each chromosome involved. SK-BR-7 presents a similar but less straightforward case. The great majority of freshly-isolated breast cancers with diploid or subdiploid karyotypes show the monosomic pattern of evolution: virtually every case falls between diploid and a line representing 1 chromosome lost per rearrangement (Dutrillaux et al, 1991). As carcinomas evolve they occasionally reduplicate their entire genome, and sometimes the resulting cells come to dominate the cancer or cell line. In subsequent evolution the chromosome number falls further, to produce near-triploid numbers. It has been suggested that the majority of breast cancers with near-triploid modes have evolved this way (Dutrillaux et al, 1991).

Our karyotypes suggest however, that near-triploid breast cancers may fall into two groups which reach a near-triploid chromosome number by two different routes: either by steadily losing chromosomes and at one point doubling their entire karyotype, or by steadily gaining chromosomes, together with acquisition of translocations. The latter may be the same as the ‘trisomic’ pattern described for near-diploid colon cancers (Muleris et al, 1988; Dutrillaux, 1995), but progressing to much greater aneuploidy. The two routes can be distinguished in many cases because the loss-with-reduplication process leads to a cell with several duplicated abnormal chromosomes, while relentless gain will tend to generate unique rearranged chromosomes that will only be paired if an extra copy of the individual abnormal chromosome is acquired. MDA-MB-157 and T-47D, for example, exactly fit the loss-with-reduplication prediction, with 9 and 7 duplicated abnormal chromosomes respectively. MDA-MB-361, on the other hand, fits the prediction of continuous gain: it has 27 out of 52 of its chromosomes rearranged, yet has no duplicated abnormal chromosomes, with the exception of two derivatives of a der(8)t(8;17)(p12;q22–25) (Morris et al, 1997; Courtay-Cahen et al, 2000). Other examples that clearly fit the continuous gain pattern are MaTu, MCF-7 and MDA-MB-435. MT-1 may have endoreduplicated from a line with a few net gains. However, not all the karyotypes fit these patterns of evolution tidily. MDA-MB-175, for example, appears to have lost chromosomes in forming most of its translocations, but at the same time to have gained extra copies of 20, 21 and 22, and the translocated 8;11.

### Are the karyotypes consistent with the published karyotypes in the literature?

Classical cytogenetic analyses of many of these lines have been reported in the literature or ATCC catalogue, but, as we and others

© 2000 Cancer Research Campaign have found, FISH results show that interpretation of banding in the highly aneuploid lines was unreliable, and reconciling FISH and previous banding results is often difficult (Morris et al, 1997; Macville et al, 1999). In several of the more complex karyotypes there were characteristic markers that matched, e.g. the hsr inserted into chromosome 11 in MDA-MB-134, and paired markers in T-47D, but in general we can only say that the data are not incompatible. For MCF-7, the mode of our sample (65, range 61–72) and some markers agree with the authentic MCF-7 samples of Osborne et al (1987). While this paper was in revision, a parallel molecular cytogenetic study of 15 breast cancer cell lines, including 7 common to our study, appeared (Kytola et al, 2000). There are significant differences in karyotype between our samples and theirs of MDA-MB-157, ZR-75-1, and, particularly, MCF-7. The lines are clearly the same: while it is possible that some discrepancies were misclassifications by SKY (not all translocations reported by Kytola et al were verified by conventional chromosome painting), most or all reflect divergence in culture.

### Cell lines that are related to each other: VP229 and VP267; MaTu and MT-1

VP229 was derived from a primary tumour, taken before any treatment, while VP267 was derived 14 months later from a local recurrence in the same patient following treatment with Tamoxifen. As expected, the two karyotypes show a common ancestral clone, and some similarities in their CGH results, but in detail they are very different, having evolved independently a great deal either in vivo or in vitro, so it would not be possible to associate any differences specifically with progression in vivo.

The karyotypes of MaTu/Ham and MT-1 have at least 6 translocations in common, so they seem to have come from the same tumour. They were also identical and heterozygous for 4 highly polymorphic dinucleotide repeats (not shown). We confirmed the authenticity of our samples by comparing them with second samples (see Methods).

Comparison of the two lines should show how clones can evolve, and again suggests that a tumour such as MT-1 that has a hypertetraploid karyotype, arises by endoreduplication of a tumour somewhat like MaTu, although sequential gain of chromosomes is also a possible explanation.

### CONCLUSIONS

The karyotypes we have described update our picture of the range and nature of translocations found in breast carcinoma cell lines, which were largely unknown because of their intractability to banding analysis. Together with the CGH data they add to the growing evidence (e.g. Wistuba et al, 1998) that the chromosome abnormalities of breast cancer cell lines are, with minor caveats, broadly representative of breast cancers, and they provide a resource for the cataloguing and analysis of translocations in these tumours. Detailed images can be found at http://www.path.cam.ac.uk/~pawefish.

### ACKNOWLEDGEMENTS

Supported by grants from the Cancer Research Campaign (to CC and PAWE) and ARC (to CT); Ligue Contre le Cancer (CB); studentships from the BBSRC and Ludwig Institute for Cancer Research (JMD), the Cambridge Commonwealth Trust Prince of Wales Scholarship, Sackler Studentship and ORS (KLG), Joshua Gilbert Rhabdomyosarcoma Fund (IR) and Ligue Contre le Cancer de I'Herault et de la Lozere (BO). We thank the cell line donors, particularly Mike O'Hare for providing many of the cell lines used and advice; and Johannes Wienberg and Malcolm Ferguson-Smith for help establishing 24-colour FISH and providing unlabelled chromosome paints.

### REFERENCES

Adeyinka A, Kytola S, Mertens F, Pandis N and Larsson C (2000) Spectral karyotyping and chromosome banding studies of primary breast carcinomas and their lymph node metastases. *Int J Mol Med* **5**: 235–240

Bautista S and Theillet C (1998) CCND1 and FGFR1 coamplification results in the colocalisation of 11q13 and 8p12 sequences in breast tumour nuclei. *Genes Chromosomes Cancer* **22**: 268–277

Bernardino J, Apiou F, Gerbault-Seureau M, Malfoy B and Dutrillaux B (1998) Characterization of recurrent homogeneously staining regions in 72 breast carcinomas. *Genes Chromosomes Cancer* **23**: 100–108

Courjal F and Theillet C (1997) Comparative genomic hybridisation analysis of breast tumors with predetermined profiles of DNA amplification. *Cancer Res* **57**: 4368–4377

Courtay-Cahen C, Morris JS and Edwards PAW (2000) Chromosome translocations in breast cancer with breakpoints at 8p12. *Genomics* **66**: 15–25

Dutrillaux B (1995) Pathways of chromosome alteration in human epithelial cancers. *Adv Cancer Res* **67**: 59–82

Dutrillaux B, Gerbault-Seureau M, Remvikos Y, Safrani B and Prieur M (1991) Breast cancer genetic evolution: I data from cytogenetics and DNA content. *Breast Cancer Res Treatment* **19**: 245–255

Fogh J and Trempe G (1975) New human tumour cell lines. In: Fogh J (ed) *Human tumor cells in vitro*, Plenum: New York, pp 115–159

Forozan F, Veldman R, Ammerman CA, Parsa NZ, Kallioniemi A, Kallioniemi OP and Ethier SP (1999) Molecular cytogenetic analysis of 11 new breast cancer cell lines. *Br J Cancer* **81**: 1328–1334

Gioanni J, Le Francois DEZ, Mazeau C, Ettore F, Lambert JC, Schneider M and Dutrillaux B (1990) Establishment and characterisation of a new tumorigenic cell line with a normal karyotype derived from a human breast adenocarcinoma. *Br J Cancer* **62**: 8–13

Hambly RJ, Double JA, Thompson MJ and Bibby MC (1997) Establishment and characterisation of new cell lines from human breast tumours initially established as tumour xenografts in NMRI nude mice. *Breast Cancer Res Treatment* **43**: 247–258

Heim S (1996) Clonal chromosome abnormalities in neoplastic cells: evidence of genetic instability? *Cancer Surveys* **28**: 9–25

Kallioniemi OP, Kallioniemi A, Piper J, Isola J, Waldman FM, Gray JW and Pinkel D (1994) Optimizing comparative genomic hybridization for analysis of DNA sequence copynumber changes in solid tumours. *Genes Chromosomes Cancer* **10**: 231–243

Kytölä S, Rummukainen J, Nordgren A, Karhu R, Farnebo F, Isola J and Larsson C (2000) Chromosomal alterations in 15 breast cancer cell lines by comparative genomic hybridization and spectral karyotyping. *Genes Chromosomes Cancer* **28**: 308–317

Macville M, Schröck E, Padilla-Nash H, Keck C, Ghadimi BM, Zimonjic D, Pospescu N and Ried T (1999) Comprehensive and definitive molecular cytogenetic characterisation of Hela cells by spectral karyotyping. *Cancer Res* **59**: 141–150

McCallum HM and Lowther GW (1996) Long-term culture of primary breast cancer in defined medium. *Breast Cancer Res Treatment* **39**: 247–259

Morris JS, Carter NP, Ferguson-Smith MA and Edwards PAW (1997) Cytogenetic analysis of three breast carcinoma cell lines using reverse chromosome painting. *Genes Chromosomes Cancer* **20**: 120–139

Muleris M, Salmon RJ and Dutrillaux B (1988) Existence of two distinct processes of chromosomal evolution in near-diploid colorectal tumors. *Cancer Genet Cytogenet* **32**: 43–50

Naundorf H, Fichtner I, Saul GJ, Haensch W and Büttner B (1993) Establishment and characteristics of two new human mammary carcinoma lines serially transplantable in nude mice. *J Cancer Res Clin Oncol* **119**: 652–656

Osborne CK, Hobbs K and Trent J (1987) Biological differences among MCF-7 human breast cancer cell lines from different laboratories. *Breast Cancer Res Treatment* **9**: 111–121

Persson K, Pandis N, Mertens F, Borg A, Baldetorp B, Killander D and Isola J (1999) Chromosomal aberrations in breast cancer: a comparison between

© 2000 Cancer Research Campaign cytogenetics and comparative genomic hybridization. *Genes Chromosomes Cancer* **25**: 115–122

Roberts I, Wienberg J, Nacheva E, Grace C, Griffin D and Coleman N (1999) Novel method for the production of multiple colour chromosome paints for use in karyotyping by fluorescence in situ hybridisation. *Genes Chromosomes Cancer* **25**: 241–250

Schröck E, du Manoir S, Veldman T, Schoell B, Wienberg J, Ferguson-Smith MA, Ledbetter DH, Bar-Am I, Soenksen D, Garini Y and Ried T (1996) Multicolor spectral karyotyping of human chromosomes. *Science* **273**: 494–497

Speicher MR, Ballard SG and Ward DC (1996) Karyotyping human chromosomes by combinatorial multi-fluor FISH. *Nature Genetics* **12**: 368–375

Stamps AC, Davies SC, Burman J and O'Hare MJ (1994) Analysis of proviral integration in human mammary epithelial cell lines immortalized by retroviral infection with a temperature-sensitive SV40 T-antigen construct. *Int J Cancer* **57**: 865–874

Telenius H, Pelmear AH, Tunnacliffe A, Carter NP, Behmel A, Ferguson-Smith MA, Nordenskjold M, Pfragner R and Ponder BAJ (1992) Cytogenetic analysis by chromosome painting using DOP-PCR amplified flow-sorted chromosomes. *Genes Chromosomes Cancer* **4**: 257–263

Tirkkonen M, Tanner M, Karhu R, Kallioniemi A, Isola J and Kallioniemi O (1998) Molecular cytogenetics of primary breast cancer by CGH. *Genes, Chromosomes and Cancer* **21**: 177–184

Veldman T, Vignon C, Schröck E, Rowley JD and Ried T (1997) Hidden chromosome abnormalities in haematological malignancies detected by multicolour spectral karyotyping. *Nature Genetics* **15**: 406–410

Wang XZ, Jolicoeur EM, Conte M, Chaffanet M, Zhang Y, Mozziconacci MJ, Feiner H, Birnbaum D, Pebusque MJ and Ron D (1999) γ-heregulin is the product af a chromosomal translocation fusing the DOC4 and HGL/NRG1 genes in the MDA-MB-175 breast cancer cell line. *Oncogene* **18**: 5718–5721

Whitehead RH, Bertoncello I, Webber LM and Pederson JS (1983) A new human breast carcinoma cell line (PMC42) with stem cell characteristics. I. Morphologic characteristics. *JNCI* **70**: 649–661

Widmaier R, Wildner GP, Papsdorf G and Graffi I (1974) Über eine neue, in vitro unbegrenzt wachsende Zellinie, MaTu, von Mamma-Tumorzellen des Menschen. *Arch Geschwulstforschung* **44**: 1–9

Wistuba II, Behrens C, Milchgrub S, Syed S, Ahmadian M, Virmani AK, Kurvari V, Cunningham TH, Ashfaq R, Minna JD and Gazdar AF (1998) Comparison of features of human breast cancer cell lines and their corresponding tumors. *Clin Cancer Research* **4**: 2931–2938

*© 2000 Cancer Research Campaign*
